# Location and hemodynamic role of perforating veins independent of saphenous veins

**DOI:** 10.1590/1677-5449.009117

**Published:** 2018

**Authors:** Carlos Alberto Engelhorn, Jheneffer Kely Soares Escorsin, Karen Christine Oliveira Costa, Larissa Miyashiro, Melissa de Morais Silvério, Raquel Cristine Gomes da Costa

**Affiliations:** 1 Pontifícia Universidade Católica do Paraná – PUCPR, Curitiba, PR, Brasil.

**Keywords:** perforating veins, ultrasonography, women

## Abstract

**Background:**

The perforating veins of the lower limbs (LL) have valves that enable the blood flow to be directed from the superficial vein system to the deep vein system and they may or may not be connected to the saphenous vein system.

**Objectives:**

To use vascular ultrasonography (VUS) to identify the frequency, location, caliber, and hemodynamic role of perforating veins that do not connect to saphenous veins, during preoperative mapping of LL varicose veins.

**Methods:**

A cross-sectional study was conducted using VUS in women to study the frequency, location, distance from the sole of the foot, and hemodynamic role of perforating veins.

**Results:**

The frequencies of perforating veins independent of the saphenous veins were 92.6%, 5.1%, and 2.3%, in the leg, thigh, and knee, respectively, and 25.2% of them were incompetent while 72.3% were drainage veins. The mean diameters of perforating veins were 2.9 mm, 3.5 mm, and 3.7 mm, in the leg, thigh, and knee, respectively. Perforating veins were located at mean distances of 23.8 cm, 43.6 cm, and 59.4 cm above the sole of the foot in the leg, knee, and thigh, respectively. Perforating veins with reflux had a mean caliber of 3.5 mm, irrespective of location.

**Conclusions:**

Perforating veins independent of the saphenous veins are most frequent in the leg, draining reflux from tributary veins. Irrespective of location, perforating veins with reflux had mean caliber of 3.5 mm.

## INTRODUCTION

 The venous system of the lower limbs (LL) comprises the deep system, responsible for 85% of venous drainage, and the superficial system, responsible for the remaining 15%. Between these two systems there are an average of 64 perforating veins, between the foot and the groin, that communicate directly or indirectly, enabling flow to drain from superficial veins to deep veins. 1

 There are four groups of perforating veins in the calf: the paratibial perforators, communicating between the great saphenous and posterior tibial veins; the perforators connecting the posterior accessory saphenous vein to the posterior tibial vein; and the lateral and anterior perforators of the leg. In the knee, the perforating veins are designated as lateral or medial, suprapatellar or infrapatellar, and popliteal fossa perforators. In the thigh, the perforators are medial, anterior, lateral, and posterior. 2

 The perforating veins drain flow from the great and small saphenous veins to the deep veins or muscular veins or drain flow from tributary veins that are independent from the saphenous vein system, and which may play different hemodynamic roles. 

 Competent perforating veins drain flow to the deep system without interfering in the caliber of the saphenous or tributary veins. Incompetent perforating veins exhibit significant reflux and may transfer this reflux to saphenous or tributary veins, causing them to dilate. Drainage perforating veins drain reflux from saphenous or tributary veins. 3

 During venous mapping with vascular ultrasonography (VUS), the medial, anterior, lateral, and posterior perforating veins of the thigh, knee, and leg should be examined. They can be identified in mode B by their course connecting superficial and deep veins and perforating the muscle fascia, thereby identifying their hemodynamic role in the venous system. 

 The objective of this study was to use VUS to identify the frequency, location, caliber, and hemodynamic role of perforating veins independent of the saphenous veins during preoperative mapping of LL varicose veins. 

## METHODS

 A cross-sectional study was conducted with women with signs or symptoms of chronic venous insufficiency (CVI) who had been referred to a vascular laboratory for venous mapping of the LL using VUS. 

 The study recruited women over the age of 18 with LL varicose veins of primary etiology and clinical classes C1 to C3 according to the clinical, etiology, anatomical and pathophysiology (CEAP) classification. The study excluded men; women with LL congenital varicose veins or varicose veins of secondary etiology or CEAP C4 to C6; and patients who had previously undergone surgical treatment for varicose veins. 

 The study was approved by the Research Ethics Commission at the Pontifícia Universidade Católica do Paraná (PUCPR), under CAAE nº 61368016.2.0000.0020 and certificate nº 111358/2016. 

### Ultrasonographic examination

 All patients included in the study were assessed using Siemens®Antares and Siemens®X 700 ultrasonography scanners with color Doppler (Issaquah, United States). 

 Deep vein system flow was assessed to exclude recent or historic venous thrombosis, with the patient in decubitus dorsal, with transverse ultrasound sweeps in B mode and maneuvers to assess vein compressibility, using a low frequency transducer (5 Mhz). 

 The great and small saphenous veins were examined with the patient standing upright, using a high frequency transducer (7 Mhz) to obtain images of the veins with longitudinal ultrasound sweeps in mode B. With the aid of color flow mapping, valve function was assessed using a manual muscle compression maneuver distal to the position of the transducer, in order to produce and detect reflux in the superficial vein system and the perforating veins. 

 Perforating veins connected to or independent of the saphenous veins were located and assessed for reflux with the patient standing upright and reflux was quantified in terms of duration of reflux exceeding 0.35 seconds. 4

 Evaluation of perforating veins independent of the saphenous veins considered location (medial, lateral, or posterior surface of the thigh, knee, and leg); height in relation to the sole of the foot; and hemodynamic role (reflux drainage, source of reflux, and competence). 

 Competent perforating veins with caliber less than 3 mm were not considered in the analysis, since they are not clinically relevant and are difficult to identify by clinical examination. 

 The results for quantitative variables were expressed as means, medians, minimum and maximum values, and standard deviations. Qualitative variables were expressed as frequencies and percentages. 

 Analysis of perforating veins, considering height from the sole of the foot, diameter, and hemodynamic role, was performed separately for each anatomic segment of thigh, knee, and leg, considering the total number of perforating veins per segment, including veins on the medial, lateral, and posterior aspects. Data were analyzed with IBM SPSS Statistics v.20. 

## RESULTS

 A total of 361 LL were analyzed in 258 women with ages ranging from 18 to 88 years (mean of 48 years): 205 right limbs (56.8%) and 156 left limbs (43.2%). 

 Of the 361 limbs assessed, 155 (42.9%) had reflux in the great saphenous vein and 93 (25.7%) had reflux in the small saphenous vein. 

 A total of 475 perforating veins independent of the saphenous veins were identified in the 361 LL assessed: 24 (5.1%) perforators in the thigh, 11 (2.3%) in the knee, and 440 (92.6%) in the leg. 

 Eighty-three (23.0%) of the 361 LL had more than one perforating vein in the same segment, breaking down as one with two perforators in the thigh and 82 limbs with two or more perforators in the leg. Sixty-three (76.8%) of the limbs with two or more perforators in the leg had two perforators, 14 (17.1%) had three perforators, five (6.1%) had four perforators, and the majority (70%) had veins in more than one location (medial, lateral, or posterior aspect). 

 In turn, 14 (58.4%) of the perforating veins in the thigh were identified in the lateral area, five (20.8%) in the medial area and five (20.8%) in the posterior area. Seventeen (70.8%) of the perforating veins in the thigh had reflux to tributary veins and seven (29.2%) drained reflux from tributary veins. All perforating veins in the thigh with diameters exceeding 3 mm were incompetent or provided drainage. 

 In relation to location and caliber ( Table 1 ), perforating veins in the thigh were a mean distance of 59.4 cm above the sole of the foot and had a mean caliber of 3.5 mm. Considering only perforating veins in the thigh with reflux, mean caliber was 3.6 mm, varying from 2.6 to 4.6 mm. 

**Table 1:** Location and caliber of perforating veins.

	**N**	**Mean**	**Median**	**Minimum**	**Maximum**	**Standard deviation**
**Thigh**						
Location (cm)	24	59.4	59	47.5	74	7.4
Diameter (mm)	24	3.5	3.5	2	4.6	0.7
**Knee**						
Location (cm)	11	43.6	43	36	53.5	5.2
Diameter (mm)	11	3.7	3.5	2.6	5.5	0.8
**Leg**						
Location (cm)	440	23.8	23	3.5	46	6.6
Diameter (mm)	440	2.9	2.8	1.6	6.3	0.7

 With regard to perforating veins in the knee, seven (63.6%) were identified in the posterior region of the knee, two (18.2%) in the medial region, and two (18.2%) in the lateral region. Six (54.5%) of the perforating veins in the knee had reflux into tributary veins and five (45.5%) drained reflux from tributary veins. All of the perforating veins in the knee larger than 3 mm were incompetent or provided drainage. 

 In terms of location and caliber ( Table 1 ), the perforating veins of the knee were located a mean distance of 43.6 cm above the sole of the foot and had a mean caliber of 3.7 mm. Considering only the perforating veins of the knee that had reflux, mean caliber was 3.8 mm, ranging from 3.2 to 4.8 mm. 

 With regard to perforating veins in the leg, 289 (65.7%) were identified in the medial region of the leg, 90 (20.4%) in the lateral region and 61 (13.9%) in the posterior region. Ninety-seven (22.0%) of the perforating veins in the leg exhibited reflux flowing to tributary veins, 330 (75.0%) drained reflux from tributary veins, and 13 (3.0%) were competent and connected to tributary veins without reflux. 

 In terms of location and caliber ( Table 1 ), the perforating veins in the leg were a mean distance of 23.8 cm above the sole of the foot and had a mean caliber of 2.9 mm. Considering only perforating veins in the leg that exhibited reflux, mean caliber was 3.4 mm, ranging from 2.0 to 6.3 mm. 

 A total of 475 perforating veins were assessed for this study, 120 (25.2%) of which exhibited reflux in the thigh, knee, or leg, with a mean caliber of 3.5 mm, ranging from 2.0 to 6.3 mm. 

## DISCUSSION

 Vascular ultrasound is the method of choice for detecting reflux of blood in specific veins, primarily using color flow mapping, which enables the extent and distribution of venous reflux to be identified with precision. 3

 Depending on study population, the incidence of sources of superficial venous reflux unrelated to the saphenous trunk veins can range from 10 to 43%. 5 ^,^ 6

 The most important sources of reflux that are not connected to saphenous veins and should be examined are the tributary veins of the posterior accessory saphenous vein, the accessory veins of the thigh, groin veins (vulvar and gluteal veins), perforating veins of the lateral and posterior surfaces of the thigh (veins of the sciatic nerve), and perforating veins of the medial, lateral, and posterior (popliteal fossa vein) aspects of the knee and leg. 5 ^,^ 7 ^-^ 11

 Our study only focused on perforating veins that are independent of the saphenous veins and identified perforating veins in the thigh, knee, and leg in 5.1%, 2.3%, and 92.6% of the limbs assessed, respectively. 

 With relation to the perforating veins of the thigh, in our study the great majority (70.8%) were incompetent and 58.3% were located in the lateral thigh. Gianesini et al. 12 assessed 2820 LL and found 26 incompetent lateral perforating veins in 24 LL, at depths of 12 to 25 mm, and connected to the femoral, deep femoral, or muscular veins. 

 In our study, just 7 (1.4%) perforating veins were identified in the posterior knee. Similarly, Delis et al. 11 assessed 818 LL and detected perforating veins of the popliteal fossa in 24 (2.9%) of the LL, and in 96% of cases there was drainage to the popliteal vein around 1.5 cm above the popliteal fold. 

 The majority (92.6%) of the perforating veins in our study were identified in the leg, 65% in the medial leg, with the primary function of draining reflux from tributary veins. Just 22% of perforating veins in the leg exhibited reflux flowing into tributary veins. It is important to highlight that competent perforating veins with calibers smaller than 3 mm were excluded from the study because they are not clinically relevant and are difficult to detect by physical examination. 

 Labropoulos et al. 13 studied 581 perforating veins in 103 limbs of 75 patients with CVI and found that 28% (163) of the veins with reflux had subfascial caliber exceeding 3.9 mm. Both competent veins and veins with reflux had smaller caliber in the lower thigh, knee, ankle, and anterior leg. 

 Sandri et al. 14 studied 500 perforators in patients with LL varicose veins, relating caliber to the probability of reflux, and observed that perforating veins with caliber greater than or equal to 3.5 mm in the thigh or leg were associated with reflux in more than 90% of cases. 

 In our study, considering only perforating veins with reflux at the thigh, knee, and leg (25%), mean calibers were 3.6 mm, 3.8 mm, and 2.9 mm, respectively. Irrespective of location, the caliber of perforating veins with reflux ranged from 2 to 6.3 mm, with mean caliber of 3.5 mm, which is similar to results from a study by Sandri et al. 14

 The authors conclude that perforating veins independent of saphenous veins are more frequent in the leg, draining reflux from tributary veins. Irrespective of location, perforating veins with reflux had a mean caliber of 3.5 mm. 
