# Supplementary material for: Location and hemodynamic role of perforating veins independent of saphenous veins
Source: J Vasc Bras. 2018 Apr-Jun;17(2):104–8. doi: 10.1590/1677-5449.009117 (PMC6205704; doi:10.1590/1677-5449.009117)
Supplement: Translation

# Translation: Localização e papel hemodinâmico das veias perfurantes independentes das veias safenas

**Authors:** Carlos Alberto Engelhorn, Jheneffer Kely Soares Escorsin, Karen Christine Oliveira Costa, Larissa Miyashiro, Melissa de Morais Silvério, Raquel Cristine Gomes da Costa

**Affiliations:** 1 Pontifícia Universidade Católica do Paraná – PUCPR, Curitiba, PR, Brasil.

**Keywords:** veias perfurantes, ultrassonografia, mulheres

## Abstract

**Contexto:**

As veias perfurantes dos membros inferiores (MMII) apresentam válvulas que permitem o direcionamento do fluxo sanguíneo do sistema venoso superficial para o profundo e podem estar conectadas ou não ao sistema das veias safenas.

**Objetivo:**

Identificar, pela ultrassonografia vascular (USV), a frequência, localização, calibre e o papel hemodinâmico das veias perfurantes independentes das veias safenas no mapeamento pré-operatório das varizes dos MMII.

**Métodos:**

Foi realizado estudo transversal utilizando a USV em mulheres com o intuito de estudar a frequência, a localização, a altura em relação à base do pé e o papel hemodinâmico das veias perfurantes.

**Resultados:**

A frequência de veias perfurantes independentes das veias safenas foi de 92,6%, 5,1% e 2,3%, na perna, coxa e joelho, respectivamente, sendo 25,2% incompetentes e 72,3% de drenagem. O diâmetro médio das veias perfurantes foi de 2,9 mm, 3,5 mm e 3,7 mm, na perna, coxa e joelho, respectivamente. As veias perfurantes localizaram-se em média 23,8 cm, 43,6 cm e 59,4 cm acima da base do pé na perna, joelho e coxa, respectivamente. As veias perfurantes com refluxo apresentaram calibre médio de 3,5 mm, independentemente da localização.

**Conclusões:**

As veias perfurantes independentes das veias safenas são mais frequentes na perna, drenando refluxo de veias tributárias. Independentemente da localização, as veias perfurantes com refluxo apresentam calibre médio de 3,5 mm.

## INTRODUÇÃO

 O sistema venoso dos membros inferiores (MMII) é composto pelo sistema profundo, responsável por 85% da drenagem venosa, e o superficial, responsável pelos 15% restantes. Entre esses dois sistemas, existem em média 64 veias perfurantes, entre o pé e a região inguinal, que fazem uma comunicação direta ou indireta, possibilitando a drenagem do fluxo das veias superficiais para as veias profundas 1 . 

 Na panturrilha são identificados quatro grupos de veias perfurantes: as perfurantes paratibiais, comunicando a safena magna e a veia tibial posterior; as perfurantes conectando o arco posterior da safena magna com a veia tibial posterior; e as perfurantes laterais e anteriores da perna. No joelho, as veias perfurantes são designadas como laterais ou mediais, supra ou infrapatelares e perfurantes da fossa poplítea. Na coxa, as perfurantes são mediais, anteriores, laterais e posteriores 2 . 

 As veias perfurantes drenam o fluxo das veias safenas magna e parva para as veias profundas ou musculares ou o fluxo de veias tributárias independentes do sistema das veias safenas, e podem desempenhar diferentes papéis hemodinâmicos. As veias perfurantes competentes drenam o fluxo para o sistema profundo sem interferir no calibre das veias safenas ou tributárias. As veias perfurantes incompetentes apresentam refluxo significativo e podem transferir esse refluxo para as veias safenas ou tributárias, causando a dilatação destas. As veias perfurantes de drenagem escoam refluxo das safenas ou tributárias 3 . 

 Durante o mapeamento venoso com ultrassonografia vascular (USV), devem ser pesquisadas as veias perfurantes mediais, anteriores, laterais e posteriores na coxa, joelho e perna, as quais são identificadas no modo B pelo seu trajeto de conexão entre as veias superficiais e profundas, perfurando a fáscia muscular, identificando o papel hemodinâmico no sistema venoso. 

 O objetivo deste trabalho foi identificar, pela USV, a frequência, localização, calibre e o papel hemodinâmico das veias perfurantes independentes das veias safenas no mapeamento pré-operatório das varizes dos MMII. 

## MÉTODOS

 Foi realizado um estudo transversal em mulheres com sinais ou sintomas de insuficiência venosa crônica (IVC), encaminhadas ao laboratório vascular para realização de mapeamento venoso dos MMII utilizando USV. 

 Foram incluídas no estudo mulheres maiores de 18 anos com varizes em MMII de etiologia primária e pertencentes às classes clínicas C1 a C3 da classificação clínica, etiológica, anatômica e fisiopatológica (CEAP). Foram excluídos homens; mulheres com varizes em MMII de etiologia secundária ou congênita ou com CEAP C4 a C6; e pacientes que foram submetidas a tratamento cirúrgico das varizes. 

 O estudo foi aprovado pelo Comitê e Ética em Pesquisa da Pontifícia Universidade Católica do Paraná (PUCPR), com CAAE nº 61368016.2.0000.0020 e comprovante nº 111358/2016. 

### Avaliação ultrassonográfica

 Todas as pacientes incluídas no estudo foram avaliadas em aparelhos de ultrassonografia com Doppler colorido Siemens^®^Antares e Siemens^®^ X 700 (Issaquah, EUA). 

 Foi realizada avaliação do fluxo no sistema venoso profundo para a exclusão de trombose venosa recente ou antiga, com a paciente em decúbito dorsal, com cortes ultrassonográficos transversais em modo B e manobras de compressibilidade das veias, utilizando-se transdutores de baixa frequência (5 Mhz). 

 O estudo das veias safenas magna e parva foi realizado com a paciente em posição ortostática, utilizando transdutor de alta frequência (7 Mhz), para a obtenção das imagens das veias em cortes ultrassonográficos longitudinais em modo B. Com o auxílio do mapeamento do fluxo a cores, avaliou-se o funcionamento valvular pela manobra de compressão muscular manual distal ao posicionamento do transdutor, a fim de produzir e detectar refluxo no sistema venoso superficial e nas veias perfurantes. 

 Foi realizada a localização e pesquisa do refluxo nas veias perfurantes conectadas ou independentes das veias safenas, com a paciente também em posição ortostática, além da quantificação do refluxo considerando o tempo de duração do refluxo superior a 0,35 segundos 4 . 

 Para a avaliação das veias perfurantes independentes das veias safenas foi considerada a localização (face medial, lateral ou posterior na coxa, joelho e perna); altura em relação à base do pé; e o papel hemodinâmico (drenagem de refluxo, fonte de refluxo e competência). 

 Para fins de análise, foram desconsideradas as veias perfurantes competentes com calibre inferior a 3 mm, por não apresentarem relevância clínica e serem de difícil identificação ao exame clínico. 

 Os resultados de variáveis quantitativas foram descritos por médias, medianas, valores mínimos, valores máximos e desvios padrões. Para variáveis qualitativas, foram apresentados frequências e percentuais. 

 A análise das veias perfurantes, considerando a altura em relação à base do pé, diâmetro e o papel hemodinâmico, foi realizada separadamente por segmento anatômico de coxa, joelho e perna, considerando a totalidade das veias perfurantes para cada segmento, incluindo as veias na face medial, lateral e posterior. Os dados foram analisados com o programa computacional IBM SPSS Statistics v.20. 

## RESULTADOS

 Foram avaliados 361 MMII de 258 mulheres com idade variando entre 18 e 88 anos (média de 48 anos), sendo 205 membros direitos (56,8%) e 156 membros esquerdos (43,2%). 

 Dos 361 membros avaliados, 155 (42,9%) apresentaram refluxo na veia safena magna e 93 (25,7%) refluxo na veia safena parva. 

 Nos 361 MMII avaliados, foram identificadas 475 veias perfurantes independentes das veias safenas, sendo 24 (5,1%) perfurantes na coxa, 11 (2,3%) no joelho e 440 (92.6%) na perna. 

 Dos 361 MMII, 83 membros (23,0%) apresentaram mais de uma veia perfurante no mesmo segmento, sendo um membro com duas perfurantes na coxa e 82 membros com duas ou mais perfurantes na perna. Dos membros com duas ou mais perfurantes na perna, 63 (76,8%) apresentaram duas perfurantes, 14 (17,1%) apresentaram três perfurantes, e cinco (6,1%) apresentaram quatro perfurantes, sendo que a maioria (70%) apresentava veias em mais de uma localização (face medial, lateral ou posterior). 

 Quanto às veias perfurantes na coxa, 14 (58,4%) foram identificadas na região lateral, cinco (20,8%) na região medial e cinco (20,8%) na região posterior. Do total de veias perfurantes na coxa, 17 (70,8%) apresentaram refluxo para veias tributárias, e sete (29,2%) drenavam refluxo de veias tributárias. Todas as veias perfurantes maiores que 3 mm na coxa eram incompetentes ou de drenagem. 

 Em relação à localização e ao calibre ( Tabela 1 ), as veias perfurantes na coxa localizaram-se em média 59,4 cm acima da base do pé, com calibre médio de 3,5 mm. Considerando somente as veias perfurantes na coxa que apresentavam refluxo, o calibre médio foi de 3,6 mm, variando entre 2,6 e 4,6 mm. 

**Tabela 1:** Localização e calibre das veias perfurantes.

	**N**	**Média**	**Mediana**	**Mínimo**	**Máximo**	**Desvio padrão**
**Coxa**						
Localização (cm)	24	59,4	59,0	47,5	74,0	7,4
Diâmetro (mm)	24	3,5	3,5	2,0	4,6	0,7
**Joelho**						
Localização (cm)	11	43,6	43,0	36,0	53,5	5,2
Diâmetro (mm)	11	3,7	3,5	2,6	5,5	0,8
**Perna**						
Localização (cm)	440	23,8	23,0	3,5	46,0	6,6
Diâmetro (mm)	440	2,9	2,8	1,6	6,3	0,7

 Quanto às veias perfurantes no joelho, sete (63,6%) foram identificadas na região posterior do joelho, duas (18,2%) na região medial e duas (18,2%) na região lateral. Do total de veias perfurantes no joelho, seis (54,5%) apresentaram refluxo para veias tributárias e cinco (45,5%) drenavam refluxo de veias tributárias. Todas as veias perfurantes maiores que 3 mm no joelho eram incompetentes ou de drenagem. 

 Em relação à localização e calibre ( Tabela 1 ), as veias perfurantes no joelho localizaram-se em média 43,6 cm acima da base do pé, com calibre médio de 3,7 mm. Considerando somente as veias perfurantes no joelho que apresentavam refluxo, o calibre médio foi de 3,8 mm, variando entre 3,2 e 4,8 mm. 

 Quanto às veias perfurantes na perna, 289 (65,7%) foram identificadas na região medial da perna, 90 (20,4%) na região lateral e 61 (13,9%) na região posterior. Do total de veias perfurantes na perna, 97 (22,0%) apresentaram refluxo para veias tributárias, 330 (75,0%) drenavam refluxo de veias tributárias, e 13 (3,0%) eram competentes e conectadas a veias tributárias sem refluxo. 

 Em relação à localização e calibre ( Tabela 1 ), as veias perfurantes na perna localizaram-se em média 23,8 cm acima da base do pé, com calibre médio de 2,9 mm. Considerando somente as veias perfurantes na perna que apresentavam refluxo, o calibre médio foi de 3,4 mm, variando entre 2,0 e 6,3 mm. 

 Do total de 475 veias perfurantes avaliadas no estudo, 120 veias (25,2%) apresentaram refluxo na coxa, joelho ou perna, com calibre médio de 3,5 mm, variando entre 2,0 e 6,3 mm. 

## DISCUSSÃO

 A ecografia vascular é o método de escolha para detectar refluxo sanguíneo em veias específicas, principalmente com a utilização do mapeamento a cores do fluxo, sendo possível identificar com precisão a distribuição e a extensão do refluxo venoso 3 . 

 Dependendo da população estudada, a incidência de fontes de refluxo venoso superficial independentes do tronco das veias safenas pode variar de 10 a 43% 5 ^,^ 6 . 

 As principais fontes de refluxo independentes das veias safenas que devem ser pesquisadas são veias tributárias do arco posterior de perna, veias acessórias da coxa, veias da região inguinal (vulvares e glúteas), veias perfurantes na face lateral e posterior (veias do nervo ciático) de coxa e veias perfurantes na face medial, lateral e posterior (veia da fossa poplítea) do joelho e perna 5 ^,^ 7 ^-^ 11 . 

 O nosso estudo focou somente nas veias perfurantes independentes das veias safenas e identificou veias perfurantes na coxa, joelho e perna em 5,1%, 2,3% e 92,6% dos membros avaliados, respectivamente. 

 Com relação às veias perfurantes na coxa, no nosso estudo a grande maioria (70,8%) era insuficiente e 58,3% localizadas na região lateral da coxa. Gianesini et al. 12 avaliaram 2820 MMII e encontraram 26 veias perfurantes laterais na coxa insuficientes em 24 MMII, com 12 a 25 mm de profundidade, conectadas com as veias femoral, femoral profunda ou musculares. 

 No nosso estudo, foram identificadas somente sete (1,4%) veias perfurantes na região posterior do joelho. Da mesma forma, Delis et al. 11 avaliaram 818 MMII e detectaram veias perfurantes da fossa poplítea em 24 (2,9%) dos MMII, apresentando em 96% dos casos drenagem para veia poplítea em torno de 1,5 cm acima da prega poplítea. 

 A maioria (92,6%) das veias perfurantes no nosso estudo foi identificada na perna, sendo 65% na região medial da perna, cuja função principal foi de drenar refluxo de veias tributárias. Somente 22% das veias perfurantes na perna apresentaram refluxo para veias tributárias. É importante destacar que veias perfurantes competentes com calibres inferiores a 3 mm foram excluídas do estudo por não apresentarem relevância clínica e serem de difícil detecção no exame físico. 

 Labropoulos et al. 13 estudaram 581 veias perfurantes em 103 membros de 75 pacientes com IVC e encontraram 28% (163) das veias com refluxo com calibre subfascial maior que 3,9 mm. Tanto as veias competentes quanto as com refluxo apresentaram menor calibre na coxa inferior, joelho, tornozelo e região anterior da perna. 

 Sandri et al. 14 estudaram 500 perfurantes de pacientes com varizes nos MMII, relacionando o calibre com a probabilidade de refluxo, e observaram que veias perfurantes com calibre igual ou superior a 3,5 mm na coxa ou perna estavam associadas a refluxo em mais de 90% dos casos. 

 No nosso estudo, considerando somente as veias perfurantes com refluxo na coxa, joelho e perna (25%) o calibre médio foi de 3,6 mm, 3,8 mm e 2,9 mm, respectivamente. Independentemente da localização, o calibre das veias perfurantes com refluxo variou entre 2 e 6,3 mm, com calibre médio de 3,5 mm, semelhante ao estudo de Sandri et al. 14 . 

 Os autores concluem que as veias perfurantes independentes das veias safenas são mais frequentes na perna, drenando refluxo de veias tributárias. Independentemente da localização, as veias perfurantes com refluxo apresentam calibre médio de 3,5 mm.
